# A unique pattern of cortical connectivity characterizes patients with attention deficit disorders: a large electroencephalographic coherence study

**DOI:** 10.1186/s12916-017-0805-9

**Published:** 2017-03-09

**Authors:** Frank H. Duffy, Aditi Shankardass, Gloria B. McAnulty, Heidelise Als

**Affiliations:** 1 Department of Neurology, Boston Children’s Hospital and Harvard Medical School, 300 Longwood Avenue, Boston, Massachusetts 02115 USA; 2 Department of Psychiatry, Boston Children’s Hospital and Harvard Medical School, 300 Longwood Avenue, Boston, Massachusetts 02115 USA

**Keywords:** Attention deficit disorder, Attention deficit/hyperactivity disorder, Autism spectrum disorder, Classification, Coherence, Connectivity, Connectome, Diagnosis, Discriminant analysis, Electroencephalogram, Medication, MRI, Principal component analysis, Spectral analysis, Split-half replication

## Abstract

**Background:**

Attentional disorders (ADD) feature decreased attention span, impulsivity, and over-activity interfering with successful lives. Childhood onset ADD frequently persists to adulthood. Etiology may be hereditary or disease associated. Prevalence is 5% but recognition may be ‘overshadowed’ by comorbidities (brain injury, mood disorder) thereby escaping formal recognition. Blinded diagnosis by MRI has failed. ADD may not itself manifest a single anatomical pattern of brain abnormality but may reflect multiple, unique responses to numerous and diverse etiologies. Alternatively, a stable ADD-specific brain pattern may be better detected by brain physiology. EEG coherence, measuring cortical connectivity, is used to explore this possibility.

**Methods:**

Participants: Ages 2 to 22 years; 347 ADD and 619 neurotypical controls (CON). Following artifact reduction, principal components analysis (PCA) identifies coherence factors with unique loading patterns. Discriminant function analysis (DFA) determines discrimination success differentiating ADD from CON. Split-half and jackknife analyses estimate prospective diagnostic success. Coherence factor loading constitutes an ADD-specific pattern or ‘connectome’.

**Results:**

PCA identified 40 factors explaining 50% of total variance. DFA on CON versus ADD groups utilizing all factors was highly significant (*p*≤0.0001). ADD subjects were separated into medication and comorbidity subgroups. DFA (stepping allowed) based on CON versus ADD without comorbidities or medication treatment successfully classified the correspondingly held out ADD subjects in every instance. Ten randomly generated split-half replications of the entire population demonstrated high-average classification success for each of the left out test-sets (overall: CON, 83.65%; ADD, 90.07%). Higher success was obtained with more restricted age sub-samples using jackknifing: 2-8 year olds (CON, 90.0%; ADD, 90.6%); 8-14 year olds (CON, 96.8%; ADD 95.9%); and 14-20 year-olds (CON, 100.0%; ADD, 97.1%). The connectome manifested decreased and increased coherence. Patterns were complex and bi-hemispheric; typically reported front-back and left-right loading patterns were not observed. Subtemporal electrodes (seldom utilized) were prominently involved.

**Conclusions:**

Results demonstrate a stable coherence connectome differentiating ADD from CON subjects including subgroups with and without comorbidities and/or medications. This functional ‘connectome’, constitutes a diagnostic ADD phenotype. Split-half replications support potential for EEG-based ADD diagnosis, with increased accuracy using limited age ranges. Repeated studies could assist recognition of physiological change from interventions (pharmacological, behavioral).

## Background

### Definitions and demographics

Attention deficit/hyperactivity disorder (ADHD) is a common childhood neurodevelopmental disorder [1], often persisting into adulthood [2, 3]. It is typically characterized by persisting patterns of pervasive inattention, impulsivity, and/or hyperactivity that frequently interfere with normal development. ADHD is often associated with functional impairments that may affect learning and academic success, interpersonal behavior, and/or overall performance [4–6].

Psychostimulant medications constitute the mainstay of ADHD treatment [7]. Fueled by the possibilities for rare but serious complications from medication [8], operant conditioning of frontal electroencephalogram (EEG) spectral content [9] is sometimes utilized as an alternative therapeutic strategy. However, the long-term efficacy of such EEG ‘neurofeedback’ therapy has not been accepted universally [10–12].

Polanczyk et al. [13] have clarified the often contradictory ADHD prevalence estimates by a thorough meta-analysis. This group demonstrated that, since the mid-1980s, the ADHD prevalence appears to have been stable at just above 5%. The many studies included in the meta-analysis demonstrated a seemingly progressively increasing incidence rate over the years – yet the analysis also showed that it resulted from the varied methodologies used in selection and identification of subjects with ADHD. For example, telephone surveys of physicians and parents resulted in much higher ADHD prevalence estimates [14] than those based upon stricter selection criteria. Additionally, the disparity in diagnostic criteria contributed to the apparently conflicting incidence reports, depending on defining ADHD/ADD as a disease versus as a symptom complex coexistent with another disease. For example, the DSM-IV disallowed a diagnosis of ADHD if the symptoms were “*… better accounted for by another mental disorder*” [15].

Implicit in the distinction between disease and symptom is the reasonable assumption that research results based upon subjects with ADHD without co-existing disease (ADHD-pure) would likely differ from results obtained from those with attention issues that are part of a larger clinical issue (ADHD-plus). The current study included representatives of both ADHD-pure and ADHD-plus categories in order to investigate neurophysiological differences and similarities.

### Neuroanatomical and neuro-functional differences in ADHD

A great many neuroimaging studies carried out during the past two decades have shown a multiplicity of structural, functional, and network differences in the brains of children and adults diagnosed with ADHD as compared to neurotypical controls. Most of these differences have focused on brain regions thought to sub-serve cognitive, motor, and attention functions. Various meta-analyses and reviews have provided comprehensive descriptions of the differences [16–18].

#### Structural MRI

Structural MRI studies using region of interest methods or automated voxel-based morphometry methods to compare children, adolescents, or adults with ADHD to neurotypically developing controls have found that overall cerebral and cerebellar volume is reduced by approximately 4–5% [19, 20] and regional volume is reduced in the prefrontal cortex, specifically orbitofrontal, superior frontal and dorso-lateral prefrontal cortices, as well as in the posterior and anterior cingulate cortex gyri, precentral gyrus, occipital cortex, limbic system (specifically the bilateral hippocampus and amygdala), basal ganglia (specifically the dorsal striatum and globus pallidus), corpus callosum (in particular, the splenium), and cerebellum (in particular, the posterior inferior vermis) [16, 21–25].

Moreover, whole and regional brain cortical gray matter thickness has been found to be reduced [22, 26, 27]. Specifically reduced are the bilateral dorso-lateral prefrontal and orbital frontal cortices, anterior and posterior cingulate cortices, and the temporo-occipito-parietal junction [27, 28]. The rate of cortical thinning in these regions appears to have a direct association with inattention and an inverse association with the severity of hyperactivity and impulsiveness [29]. Furthermore, reductions in anterior cingulate cortex gray matter volume were correlated with selective inattention scores and changes in the networks within and between the prefrontal cortices, and the striatum and cerebellum were correlated with cognitive impairments such as distractibility, forgetfulness, impulsivity, poor planning, and locomotor hyperactivity in children and adults with ADHD [21, 30, 31].

Employing diffusion tensor imaging, Yoncheva et al. [32] found that the greatest ADHD differences in adults and children were primarily limited to the ‘mode of anisotropy’ , which is sensitive to crossing fibers.

#### Functional MRI

Functional MRI studies have found abnormal connectivity patterns across several brain regions in ADHD, particularly the frontal cortex. In children or adults with ADHD, abnormal patterns of functional activation have been found in the orbital, dorso-lateral and mesial regions of the prefrontal cortex as well as in premotor and motor regions, the orbital frontal cortex, which is associated with social inhibition and impulse control [33], the dorso-lateral prefrontal cortex, which is associated with planning, working memory and attention processes [34], and in the bilateral inferior prefrontal cortices. Moreover, reduced functional connectivity has been found between right inferior fronto-frontal, fronto-striatal, and fronto-parietal neural networks specifically during a stop and switch task [35].

Functional activation is significantly decreased in multiple brain regions in ADHD during several cognitive performance tasks and in resting-state [16, 36, 37]. These regions include the cingulo-fronto-parietal network involving its fronto-striatal and fronto-parietal pathways, as well as in the dorso-lateral and ventrolateral prefrontal cortex, and in the superior parietal cortex. During attention tasks studies, Bush [16] also found hypoactivation of the dorsal anterior cingulate cortex (dACC), which is strongly associated with the processes of attention, target detection, novelty detection, response selection, response inhibition, error detection, and motivation [33]. During the resting state decreased connectivity was found between the dACC-posterior cingulate [38, 39] and dACC [40, 41], and between thalamus and basal ganglia areas (in particular, putamen) [42], while increased connectivity was found between the dACC and the bilateral thalamus, bilateral cerebellum, and bilateral insula [43].

During a visual sustained attention task, Li [44] reported significantly reduced regional activations in the bilateral thalami (in particular, the pulvinar nuclei), significantly decreased functional connectivity between bilateral pulvinar and right prefrontal regions, and significantly increased connectivity between the right pulvinar and the bilateral occipital regions. In ADHD subjects studies have found significantly decreased functional connectivity among the brain regions that form the default mode network (the network of brain regions that is more active during rest than during tasks demanding sensory and cognitive processing) and between putamen and thalamus [42, 45]. The incremental task-related deactivation of the default mode network regions have been associated with increased task engagement as well as transitions from rest-to-task states [46, 47].

Thus, there are substantial MRI-based data that indicate widespread brain differences between ADHD and neurotypical controls. This body of information is of potential importance in understanding the basic underpinnings of this common and clinically vexing disability. Recently, the ADHD-200 Consortium undertook the feasibility of ‘breaking’ the reliance of psychiatry and behavioral neurology upon the classic ADHD classification system (based upon clusters of symptoms) by supplanting this with MRI derived measurements [48]. The Consortium completed a study, fueled by the availability of a large set of MRI-based imaging data and the efforts of multiple investigators at multiple sites, to blindly classify a ‘test set’ of mixed normal and ADHD subjects’ MRI data after preliminary evaluation of an also-supplied and openly identified ‘training set’ of similarly mixed but identified normal and ADHD subjects. At study conclusion it was determined that “*The average prediction accuracy was 49.8% (range: 37.4–60.5%)*” [48]. One competing group explored predictive classification based solely upon supplied demographic/phenotypic variables and achieved a prediction rate accuracy of 62.5%. The consortium concluded “*…that diagnostic assessment cannot currently be based on structural or functional brain imaging, nor do we believe that brain imaging will ultimately result in a first-line tool in clinical psychiatry. The costs of conducting brain imaging … would be prohibitive*” [48].

#### EEG spectral analysis

Comparative studies between ADHD and neurotypical control subjects using EEG have primarily used traditional power spectral analysis of individual channels and frequencies. The most consistent finding, especially among practitioners of EEG neurofeedback, has been an increased power in the theta band (4–7 Hz) and decreased power in the alpha and beta bands (10–30 Hz) [49–52]. An analysis of the clinical utility of theta found that the theta power increase differentiated between ADHD and control subjects with a 62% accuracy, and that a significantly elevated theta characterized a subgroup of ADHD patients and was significantly correlated with inattention and executive problems [53]. Others have evaluated EEG spectral content during a large number of differing cognitive tasks such as Stroop, go/no-go, reading, and drawing [54–61]. Here, results are less consistent perhaps reflecting the diverse testing paradigms employed.

#### EEG measures of approximate entropy (ApEn)

According to Sohn et al. [61], ApEn is “*…an index that quantifies the irregularity or complexity of a dynamical system. It is particularly effective with short and noisy time-series data*”, such as EEG. The authors compared subjects with ADHD to normal controls at rest and then again during a continuous performance task. Between groups difference in complexity and spectral content was noted over the right frontal electrodes but only during the continuous performance task. The authors concluded that “…*that cortical information processing is altered in ADHD adolescents, and thus their levels of cortical activation may be insufficient to meet the cognitive requirements of attention-demanding tasks*” [61]. These findings suggest that signal processing techniques beyond spectral analysis and its derivatives may prove useful in understanding the complexities of ADD. ApEn is not utilized in the current study; its potential utility is to be explored in the future.

#### EEG coherence

Given the MRI evidence reviewed above demonstrating altered brain networks and connectivity for ADD subjects when compared to neurotypical controls, such comparisons utilizing EEG coherence have also been undertaken. EEG spectral coherence, on a frequency by frequency basis, represents the consistency of the phase difference between two EEG signals when compared over time [62]. According to Srinvasan et al. [63], coherence is a measure of synchronization between two EEG signals based on phase consistency. While two signals may have different phases, high coherence occurs when this phase difference tends to remain constant. In each frequency band, coherence measures whether two signals can be related by a linear time invariant transformation, i.e., a constant amplitude ratio and phase shift (delay). In practice, EEG coherence depends mostly on the consistency of phase differences between channels. High coherence values are taken as a measure of strong connectivity between the brain regions that produce the compared EEG signals [64].

Several studies utilizing EEG coherence have shown significant differences between control and ADHD subjects as well as differences within the ADHD population in regard to the degree of response to therapeutic medications [65–67]. Murias et al. [68], using high density EEG recordings, summarized that, by use of EEG coherence, it could be shown that “…*altered functional connectivity, particularly among frontal regions, is implicated in ADHD*”.

In a recent comprehensive paper, Helgadottir et al. [69] utilized EEG spectral coherence measures to classify a large population of control and ADHD subjects. Utilizing a training test set cross-validation process, the authors identified consistent group differences that were stronger when age was factored into the analyses. The most relevant coherences among the 12 electrode pairs chosen for analysis were noted to involve left-right electrode pairs C3-C4 (central) and T7-T8 (temporal). The authors suggested that their study “…*demonstrates that an EEG-based method using classification algorithms can bring a new perspective to the diagnosis of ADHD in children and adolescents* …” [69]. They also suggested that EEG-based classification algorithms may enable monitoring of subjects longitudinally.

### Aims of the current study

The main aim of the current study was to assess the validity of EEG coherence as a means to differentiate subjects with ADHD from healthy neurotypical control subjects. In order to achieve this, a scientifically and technically sound and comprehensive study of such subjects was designed following the step-wise goals outlined below:

1. To identify a population of subjects with attention disorder within an existing large EEG database and to select age-comparable neurotypical control subjects from the same database.
2. To base analyses on a measure of connectivity (EEG coherence) between and among a full set of 24 standard electrodes (Fig. 1), that includes sub-temporal electrodes.

**Fig. 1:**
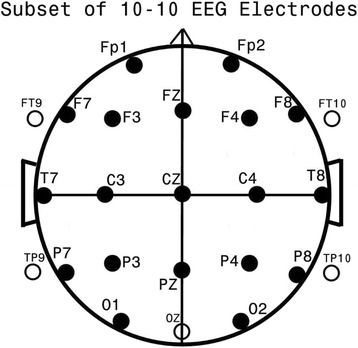
Standard 24 EEG electrode names and positions. Head in vertex view, nose above, left ear to left. The ‘standard’ 19, 10–20 electrodes are shown as black circles. EEG electrodes: Z: Midline; FZ: Midline Frontal; CZ: Midline Central; PZ: Midline Parietal; Even numbers, right hemisphere locations; odd numbers, left hemisphere locations; Fp: Frontopolar; F: Frontal; C: Central; T: Temporal; P: Parietal; O: Occipital. An additional subset of five, 10–10 electrodes are shown as open circles. EEG electrodes: FT: Frontal-Temporal; TP: Temporal-Parietal; OZ: Midline Occipital. FT and TP electrodes are often referred to as ‘subtemporal’ electrodes
3. To avoid analysis of high frequency beta and gamma EEG spectral bands owing to the well-known, strongly confounding, impact of muscle activity on these bands [70, 71].
4. To base analyses on awake, resting EEG thereby avoiding the possibility for group-specific, task-based artifact associated, in our experience, with behavioral paradigms that require response(s).
5. To undertake a rigorous, previously-described process minimizing any effects of EEG artifact upon analytic results [62].
6. To produce and analyze the full matrix of all possible EEG coherence channels, i.e., each electrode’s connectivity to all other electrodes, across all individual EEG spectral bands.
7. To reduce the large resulting coherence variable number by the use of principal component analysis (PCA), instead of by a priori ‘knowledge based’ preselection, thereby being guided by the actual structure of the coherence data implicit in the unrestricted, large omnibus set of all possible coherences.
8. To explore the potentially confounding effects of medications and coexisting diagnoses upon detection of subjects with attention disorder by coherence-based measures.
9. To explore result consistency by means of multiple split-half replications.
10. To explore age sub-grouping and compare and contrast to whole population results.
11. To determine whether a pattern of coherence difference manifests a recognizable EEG coherence pattern or ‘connectome’ specific to attention disorder, and to explore its possible clinical relevance.

## Methods

### Study population

All analyses were performed at Boston Children’s Hospital (BCH), a Harvard Medical School affiliated teaching hospital in Boston, Massachusetts, USA. The Developmental Neurophysiology Laboratory (DNL), within the Department of Neurology at BCH, is under the direction of the first author. The DNL maintains an extensive database of patients and research subjects including digitized, unprocessed (raw) EEG data, accompanied by comprehensive referral information, for thousands of patients and research subjects including neurotypical control group subjects. Patients are typically referred to rule out epilepsy by studies that incorporate lengthy digital EEG recordings. Research subjects also include similarly collected EEG data obtained from those subjects selected to serve as neurotypical controls (Table 1).

**Table 1:** Populations studied

Description	Total	Control	Attention Deficit Disorder
Fulfilling criteria, used for principal component analysis and full group discriminant	966	619	347
Ages 2–22 years, % female		15	13
Subgroups by age			
2–8 years	327	221	106
8–14 years	517	348	169
14–22 years	122	50	72

#### Patients with ADD

From the DNL database, patients were identified on the basis of referrals from experienced BCH and other Harvard Medical School affiliated teaching hospital clinicians, including neurologists, psychiatrists, and psychologists, who identified patients as having ADD or ADHD as their primary clinical problem. Referral for clinical EEG was initiated to rule out the possibility that attention lapses might reflect a covert manifestation of epilepsy.

Necessary inclusion criteria thus required a referral diagnosis of ADD or ADHD (hereinafter combined and referred to as ADD). Diagnosis relied upon DSM-IV or DSM-5 criteria sometimes supplemented by one or more rating scales (e.g., Connors [72, 73]) and/or augmented by the clinical opinion of one or more expert behavioral neurologists based upon clinical history and examination.

Additional information regarding etiology, academic performance, concurrent behavioral or mood issues, sensorimotor problems, medication(s), and other comorbidities were available for all subjects. This allowed for definition of population subgroups in order to explore the putative impact of comorbidities and medication upon ADD neurophysiology (see Table 1 and Results).

#### Healthy neurotypical control group subjects

From a population of healthy children previously recruited and studied at the Neurobehavioral Infant and Child Studies Lab, affiliated with the DNL, for various neuro-developmental research projects, a group of neurotypical control subjects was obtained from the DNL database in order to provide a comparison group of children, selected to be normally functioning while avoiding comparison with an exclusively ‘super-normal’ group.

Necessary inclusion criteria were as follows: (1) for younger subjects, living at home and as indicated, enrolled at regular school, and considered normal by their parents; or (2) for older subjects, gainfully employed and/or enrolled in college or the equivalent, and identified as functioning within the normal range on standardized developmental and/or neuropsychological assessments performed during the respective research studies.

Exclusion criteria were as follows: (1) diagnosed neurologic or psychiatric illness such as ADD/ADHD, mood disorder, autism, psychosis, global developmental delay, genetically based syndrome(s), significant head injury, drug dependency, or currently active seizure disorder; (2) abnormal neurological examination as identified during the research study; (3) an EEG report suggesting an active seizure disorder or epileptic encephalopathy (note that subjects with rare EEG spikes or EEG ‘normal variants’ were not excluded); (4) noted by the research psychologist and/or experienced EEG technologist to have significant attention, hyperactive, psychotic, or autistic features; (5) newborn period diagnoses of intraventricular hemorrhage, retinopathy of prematurity, hydrocephalus, cerebral palsy or other significant conditions likely influencing EEG data; and/or (6) receiving medication treatment at the time of the EEG study.

### Measurements and data analysis

#### EEG data acquisition

EEG technologists, naïve to the study’s goals, and specifically trained and skilled in working with children, adolescents and young adults within the study’s age group and diagnostic range, obtained EEG data in one of three ways. The primary method involved the use of up to 32 gold-cup scalp electrodes affixed with Collodion or an equivalent after measurement. Analyses of these data were subsequently restricted to the following 24 channels available for all subjects: FP1, FP2, F7, F3, FZ, F4, F8, T7, C3, CZ, C4, T8, P7, P3, PZ, P4, P8, O1, OZ, O2, FT9, FT10, TP9, TP10 (Fig. 1). Data were primarily obtained from Grass™ (Grass Technologies Astro-Med, Industrial Park 600, East Greenwich Avenue, West Warwick, RI 02893 USA) EEG amplifiers with 1–100 Hz bandpass filtering and digitized at 256 Hz for subsequent analyses. More recent subjects’ EEG data were obtained with Neuroscan™ (Compumedics Neuroscan, 6605 West W.T. Harris Boulevard, Suite F, Charlotte, NC 28269 USA) or EGI™ (Electrical Geodesics Inc., 1600 Millrace Drive, Suite 200 Eugene, OR 97403 USA) amplifiers that utilized a higher spatial (more electrodes) and/or higher temporal (1–500 Hz) resolution. Such ‘high resolution’ data were adjusted to conform to the characteristic parameters of the Grass amplifiers. The EGI electrode nets were utilized with conductive paste, as saline soaked electrodes appear to promote electrode ‘bridging’ , which in turn artificially alters spectral coherence between bridged electrodes. Photogrammetry™ was employed to establish electrode location when high electrode density nets were applied without direct measurement of electrode location. After photogrammetry, reduction to 24-electrode location was accomplished with BESA™ (BESA GmbH, Freihamer Strasse 18, 82116 Gräfelfing, Germany) software by 3D spline interpolation. Spectral band pass differences were equalized by in-house developed software utilizing forward and reverse Fourier transforms [74].

For all subjects, EEG data were gathered in the eyes closed, waking state. Adequate periods of waking EEG were assured for collection. ‘Times out’ to relax and regain composure were offered as indicated. EEG data collected during epochs of evoked potential formation to visual or auditory stimulation were excluded from analysis in the current study.

#### Measurement issues and solutions

EEG studies are confronted by three major methodological problems. The first involves management of abundant artifacts resulting from eye movement, eye blink, poor electrode-scalp contact, drowsiness, and/or muscle activity, all of which may be prominent in younger and/or more behaviorally difficult to manage children such as those with ADD. It has been well established that even EEGs appearing ‘clean’ by visual inspection may yet contain significant artifacts [75, 76]. Artifact may provoke excessive variance and mask discovery of group difference and/or may be group specific and thereby promote appearance of spurious group differences [77]. Second is capitalization upon chance from application of statistical tests on the basis of too many collected/analyzed variables with subsequent chance findings that spuriously support an experimental hypothesis (Type 1 or false positive error [78]). Third is failure to find valid group differences resulting from a priori variable reduction in order to avoid capitalization upon chance, which may involve discarding of variables that manifest true group differences (Type 2 or false negative error [78]). Methods discussed below were designed to specifically address these three common methodological/analytic problems confronting all EEG-based data analyses.

#### Artifact management

As previously outlined in greater detail [62] the following steps were instituted for artifact management:

1. EEG segments containing obvious movement artifact, electrode artifact, eye blink storms, drowsiness, epileptiform discharges, and/or bursts of muscle activity were marked for removal from subsequent analyses by expert visual inspection (initially by the EEG technologist with subsequent second review by the first author, an experienced clinical electroencephalographer). Artifact identified in a subset of channels resulted in removal of all channel data for the duration of the artifact.
2. EEG data were subsequently filtered below 50 Hz with an additional 60 Hz mains filter.
3. Remaining ambient eye blink was removed by utilizing the source component technique [79], as implemented in the BESA software package. These combined techniques resulted in EEG data that appeared largely artifact free, with rare exceptions of low-level temporal muscle fast activity and persisting frontal and anterior temporal slow eye movement artifacts, which nonetheless remain capable of contaminating subsequent analyses.
4. A regression analysis approach [80] was employed to remove the remaining potential contaminants from subsequently created EEG coherence data (see below). Representative frontal slow EEG spectral activity, taken to reflect residual eye blink, and representative frontal-temporal EEG spectral fast activity, taken to represent residual muscle artifact, were used as independent variables within multiple regression analysis, where coherence data variables (see below) were treated as dependent variables. Residuals of the dependent variables, now uncorrelated with the chosen independent artifact variables, were used for the subsequent analyses.

#### Calculation of spectral coherence and spectral variables

As previously described [62], 8–20 minutes of eyes closed, awake state EEG data per subject were transformed within BESA to the Laplacian or current source density reference. This approach provided ‘reference-independent’ data that are primarily sensitive to underlying cortex and relatively insensitive to deep/remote EEG sources. Use of the current source density also reduces spurious effects of volume conduction upon coherence by emphasizing sources at small spatial scales [63] and is optimal for coherence analyses. The current source density reference technique is considered superior to the use of the common average reference for studies involving spectral coherence [63].

Spectral coherence was calculated using a Nicolet™ (Nicolet Biomedical Inc., 5225 Verona Road, Madison, WI 53711 USA) software package, according to the conventions recommended by van Drongelen [64] (p. 143–4, equations 8.40, 8.44). In practice, coherence is typically estimated by averaging over several epochs or frequency bands [64]. In the current project, a series of 2-sec epochs was utilized to process available EEG segments. Spectral coherence measures were derived from the 1–32 Hz range, in 16, 2-Hz wide spectral bands, resulting in 4416 unique coherence variables. The diagonal of the 24 by 24 electrode coherence matrix has a coherence value of 1 – each electrode to itself; and half of the 552 remaining coherence values are duplicates of the other half. This results in 276 unique coherences per spectral band. Multiplication by the 16 spectral bands in turn results in 4416 unique spectral coherence values per subject. Standard spectral data were calculated, using the common average reference, by Fast Fourier Transform over the same frequency range noted above and based upon the Fast Fourier Transform algorithm described in Press et al. [74] (p. 411–2). Resulting spectral data were solely utilized in order to approximate residual artifact contamination (see Artifact Management) and facilitate removal by regression analysis.

#### Prevention of capitalization upon chance: variable number reduction by creation of coherence factors

In order to avoid capitalization on chance resulting from the use of too many variables and to facilitate subsequent statistical analysis, PCA of the EEG coherence data was employed as an objective technique to reduce variable number meaningfully whilst preserving information content [62, 81]. The coherence data were first normalized (centered and shifted to have unit variance) so that resultant factors reflected deviations from the average. In order to avoid loss of sensitivity by a priori data limitation, an unrestricted form of PCA [82] was applied allowing all coherence variables per subject to enter analysis. By employment of an algorithm based upon singular value decomposition [74, 83], a data set of uncorrelated (orthogonal) principal components or factors [81, 82] was developed in which the identification of a small number of factors following Varimax rotation [84] describe an acceptably large amount of variance [85]. Varimax rotation enhances factor contrast yielding higher loadings for fewer factors whilst retaining factor orthogonality. Although not the only PCA method applicable to large, asymmetrical matrices (4416 variables by 966 cases as in the current study), singular value decomposition may be used to solve under-determined and over-determined systems of linear equations [74]; it is among the most efficient techniques used for PCA [82]. This approach to variable number reduction has been successfully used in prior studies of EEG spectral coherence in infants [86] and adults [82, 87, 88], children with autism [62, 89], and pediatric and young adult subjects with schizophrenia [90]. When total population size is over 200, as in the current study, coherence factor formation consistency by split-half replication becomes redundant (unpublished finding).

#### Discrimination of subject groups by use of EEG spectral coherence variables

BMDP™-P7M (stepwise discriminant functional analysis) facilitates several analyses of importance to our project. First, when discriminant functional analysis completes formation of stepwise variable selection it creates the Wilk’s lambda measure, which can be approximated by an F value as for a one-way ANOVA. This gives an indication of classification success at an early point of the analytic process. Second, all classification functions and outcome indices can be formed on a designated ‘training-set’ containing at least two subject groupings; results may be assayed on a designated ‘test-set’ that has been specifically left out of the initial analytic processes. Third, for classification of each training-set subject, P7M computes the Mahalanobis distance measure to the group mean of each training-set population as well as the posterior probabilities belonging to each group. Accordingly, subjects are classified into one or the other training-set group on the basis of the highest posterior probability, i.e., the smallest Mahalanobis group distance. Success of training-set generated classification can be assayed by the set aside test-set subjects’ classification success. Fourth, P7M provides a new continuous, canonical variable derived from a calculated linear combination of input variables that best discriminates between the two groups under study. Although the rules used to form the canonical variable are generated from the designated training-set population data, these rules can also be used to directly calculate canonical variable scores for members of a secondary test-set. In this instance, univariate analysis of the test-set canonical variable between members of test-set subject groups, or between the control population and a single left out population, can provide another estimate of prospective classification success. For example, two-group t-tests (BMDP-3D) can be performed utilizing the canonical discriminant variable produced by a training-set test on the corresponding test-set population. Fifth, P7M encompasses a random number generator that can be used to randomly split a large population into multiple training-sets and test-sets to assess prospective classification success, e.g., split-half replication, which is best done when total population size is relatively large. Sixth, P7M provides a simpler prospective classification success estimate referred to as jackknifing or leave-one-out [91, 92], where a single subject test-set is formed and classification success is recorded using the full population, minus the left out subject, as the training-set. This process is repeated until all subjects have been individually left out. Jack-knifing is preferred to estimate prospective classification success when total population size is relatively small.

#### Factor description; relationship of PCA outcome factors to input coherence variables

Individual outcome factors were formed as linear combinations of all input variables with the weight or loading of each coherence variable upon a particular factor as determined by the PCA computation [93]. Meaning of outcome factors was discerned by inspection of the loadings of the input variables upon each individual factor [81, 93]. Factor loadings were treated as if they were primary neurophysiologic data and displayed topographically [94, 95]. The highest 10–15% of coherence loading values, are displayed as previously utilized [62, 86–90], in order to facilitate an understanding of the meaning(s) of individual factors, as shown in Fig. 3. In this figure, each head image shows the top or highest set of coherences that load upon an individual factor at the indicated frequency or frequency range. Taking into account the sign (±) of the coherence loading upon the PCA derived factor, the sign (±) of the factor’s loading on the group study discriminant function, and the directional sign (±) of the two groups (CON vs. ADD) plotted upon the discriminant function axis it is possible to infer the direction of coherence difference for the ADD related group for an individual factor. Red lines signify increased coherence and yellow lines decreased coherence for the ADD group (for the given factor). For example, Factor 12 of Fig. 3 illustrates decreased 8–18 Hz posterior (largely occipital-temporal) coherences in the ADD population. In short, the lines delineate coherences primarily associated with a factor and the line color delineates coherences that are increased or decreased in ADD for each factor.

## Results

### Neurotypical subjects (CON)

**Fig. 2:**
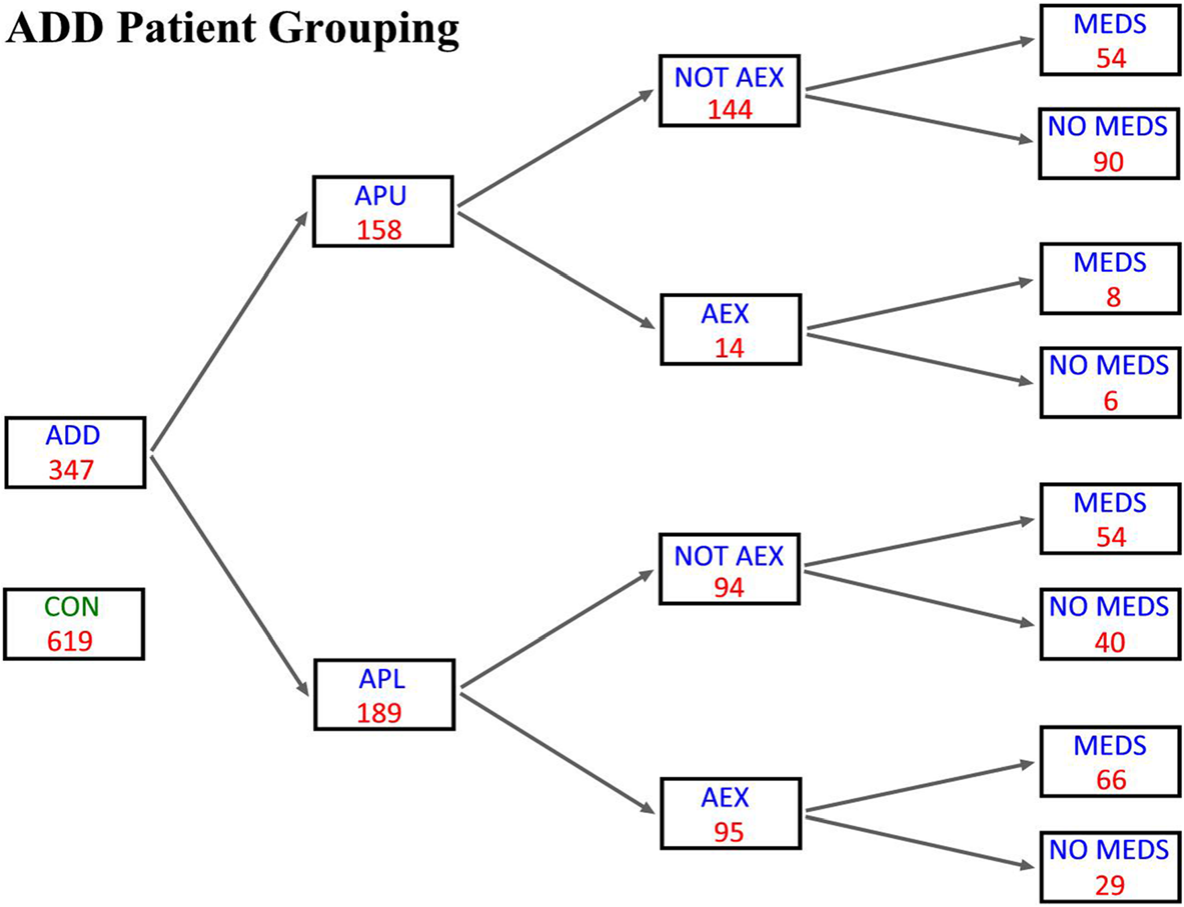
Subject groupings. The entire population consists of 966 subjects, 347 as attention deficit disorder (ADD) and 619 as control. Breakdown of the ADD ‘pure’ (APU) population is shown above and ADD ‘plus’ (APL) population below

A total of 619 control subjects with available EEG data, who fulfilled the defined criteria for the CON group and fell within the 2–22 year age range, were identified within the Neurobehavioral Infant and Child Studies Lab database (Table 1 and Fig. 2).

### ADD subjects

A total of 347 ADD subjects, with available EEG data, who met criteria for the ADD group and fell within the 2–22 year age range were identified within the DNL database (see Table 1, and Fig. 2 for subgroupings). On the basis of available clinical information, those taking psychoactive medications within the ADD group were identified as medicated (subcategory MED); a total of 182 ADD subjects were identified as MED.

All ADD subjects, including the MED group, were also divided into the subcategories APU (ADD ‘pure’) and APL (ADD ‘plus’). APU subjects had ADD as their primary medical diagnosis with no obvious causal factor or medical comorbidity. Mild school problems and occasional extremes of behavior were accepted within the APU subgroup. APU subjects were allowed to have questioned but unconfirmed or reports of mild additional learning, language, mood, anxiety, sensory integration, or motor planning disorders.

Subjects entered the APL group when the possibilities for potentially relevant comorbidities were raised including ‘autistic-like’ behavior, remote but mild closed head injury, past history of seizures no longer active, occasional mood swings without firm diagnosis of bipolar disorder, and previous drug abuse.

The final category represented those subjects whose adjunctive symptomatic history or prescribed medication use was more ‘extreme’. This more historically deviant group is referred to as AEX (ADD, extreme). AEX encompasses subjects taking psychoactive medications outside of the category usually employed for treatment of otherwise uncomplicated ADD and/or with compelling history suggestive of underlying causative disease(s). It includes subjects where attentional issues remained the primary complaint but who also carried confirmed additional diagnosis such as epilepsy, bipolar disorder, significant (etiologically relevant) head injury or encephalitis, and/or a diagnosis of developmental delay. All ADD subjects were categorized as APU or APL and any one subject might also fall into categories AEX and/or MED. Such coarse subcategorizations were formed to assist the determination of whether physiological difference between CON and ADD groupings might be complicated by the presence of the potentially confounding historical details; Figure 2 graphically displays the entire utilized population. The sequence APU to APL to AEX reflects progressive complexity of medical history but does not necessarily reflect an augmented degree of attentional disorder.

### Generation and selection of spectral coherence variables

#### Results of PCA

All available 619 CON and 347 ADD subjects were combined and utilized for singular value decomposition-based PCA on a 4416 coherence variable by 966 case data matrix. Distribution of variance among output coherence factors demonstrated a satisfactory condensation into a small number of factors – 795 factors described 99.01%, 365 factors described 90.03%, 40 factors described 50.39%, 7 factors described 24.90%, and the first factor alone described 6.43% of the total variance after Varimax rotation. The first 40 varimax rotated factors, describing just over half of the total variance, were taken to constitute each subject’s EEG coherence data for subsequent statistical analyses. The only investigator intervention in this process involved selection of the PCA data reduction protocol and the decision to utilize as many created factors (in this case 40) as needed to describe at least half of the information (variance) contained within the original coherence variables. Resulting ‘unbiased’ data reduction was on the order of 4416:40 or 110:1. Given the extensive subject age range, multivariate regression (BMDP-6R) was used to remove an age effect from these 40 coherence factors and the 40 factor age-regression ‘residuals’ were utilized to represent subjects for subsequent analyses.

CON versus ADD two-group F test results showed that 17 of the 40 individual factors were significant at a significance level better than *P* ≤ 0.05 (Table 2). Ten were better than the *P* ≤ 0.01 level and seven were better than the *P* ≤ 0.0001 level. Note the highest F value of 376.69 for top ranked Fac13 is nearly nine times the F value for the second ranked Fac12 at 39.87.

**Table 2:** Factor ranking, group CON vs. group ADD by F-test, 17 of 40 factors with *P* ≤ 0.05

Rank	Factor	F to enter	*P*	df
1	Fac13	376.69	0.0001	1964
2	Fac12	39.87	0.0001	1964
3	Fac4	38.43	0.0001	1964
4	Fac1	37.87	0.0001	1964
5	Fac2	23.43	0.0001	1964
6	Fac3	17.59	0.0001	1964
7	Fac9	15.70	0.0001	1964
8	Fac28	13.96	0.0002	1964
9	Fac27	9.01	0.0028	1964
10	Fac11	8.74	0.0032	1964
11	Fac7	6.58	0.0105	1964
12	Fac35	6.41	0.0115	1964
13	Fac40	6.00	0.0145	1964
14	Fac8	5.38	0.0208	1964
15	Fac15	4.93	0.0206	1964
16	Fac34	4.32	0.0379	1964
17	Fac17	4.07	0.0438	1964

#### Significance tests of CON versus ADD

Two trials were taken to assess the multivariate statistical significance between the CON and ADD populations when represented by all 40 coherence derived factors. The first trial involved discriminant analysis of the 619 CON versus the entire (n = 347) ADD population with all 40 factors forced to enter. The discrimination was successful at the *P* ≤ 0.0001 level by Wilk’s lambda. The classification success for both groups was high (CON 88.5%, ADD 94.5%), indicating that the 40 factors, as a group, are significantly different between the CON and ADD groups.

The second trial was chosen to represent a comparison between all 619 controls and the most unambiguous ADD representatives – 90 APU subjects not taking medications and not within the AEX subgroup (Fig. 2). This cleanest of the APU population is referred to as APUU. P7M was utilized with all 40 factors forced-in. This CON-APUU group comparison was significant at *P* ≤ 0.0001 by Wilk’s lambda. The classification success for both groups was 91.1%. These findings indicate that the 40 factors, as a group, are also significantly different between the CON and APUU groups. Of interest, the passively classified remaining 257 ADD subjects (not involved in the discriminant creation, treated as a test set) were correctly classified 87.55%. Thus, this additionally indicated that classification rules developed on ADD pure subjects may correctly classify ADD subjects on medications and/or additional complicating clinical factors. This possibility is explored more thoroughly below and with variable selection/stepping allowed.

#### Classification of medicated ADD subjects on the basis of non-medicated ADD subjects

The discriminant was formed on CON (n = 619) versus all non-medicated ADD (n = 165) subjects. The CON subjects were 88.9% and the non-medicated ADD were 90.9% correctly classified on the basis of 25 factors where stepping was permitted. The excluded, medicated ADD subjects (MED; n = 182) were 88.46% correctly classified. Thus, classification rules generated on the basis of unmedicated ADD subjects can successfully classify left-out medicated ADD subjects with high accuracy. The t-test of the discriminant function variable, created by contrasting the CON versus unmedicated MED subgroup, was very significant (*P* ≤ 0.0001) when passively assessed between the CON and the left-out MED population (Table 3.1).

**Table 3:** Classification of left-out clinical groups

1. Classification of medicated ADD (MED) on basis of CON vs. unmedicated ADD (UMED)								
Training Set: CON vs. UMED				Test Set: MED				
Num CON	% CON	Num UMED	% UMED	Num MED	% MED	DFA significance		
correct	correct	correct	correct	correct	correct	t	df	*P*
550/619	88.9%	150/165	90.9%	161/182	88.46%	25.55	799	0.0001
Top 5 FAC: 13, 12, 4, 1, 2								
2. Classification of ADD plus (APL) on basis of CON vs. ADD pure (APU)								
Training Set: CON vs. APU				Test Set: APL				
Num CON	% CON	Num APU	% APU	Num APL	% APL	DFA significance		
correct	correct	correct	correct	correct	correct	t	df	*P*
556/619	89.8%	141/158	89.2%	164/189	86.77%	24.04	806	0.0001
Top 5 FAC: 13, 12, 4, 2, 3								
3. Classification of ADD extreme (AEX) on basis of CON vs. ADD not extreme (nAEX)								
Training Set: CON vs. nAEX				Test Set: AEX				
Num CON	% CON	Num nAEX	% nAEX	Num AEX	% AEX	DFA significance		
correct	correct	correct	correct	correct	correct	t	df	*P*
554/619	89.5%	219/238	90.2%	98/109	89.90%	20.33	726	0.0001
Top 5 FAC: 13, 12, 4, 2, 1								

*CON* neurotypical controls, *DFA* discriminant function analysis, *df* degrees of freedom, *FAC* factor(s), *t* Student’s t-test, *P* probability

#### Classification of APL subjects on the basis of APU subjects

The discriminant was formed on CON (n = 619) versus APU (n = 158) subjects. The CON subjects were 89.8% and the APU were 89.2% correctly classified on the basis of 20 factors where, again, stepping was permitted. The excluded APL subjects (n = 189) were 86.77% correctly classified. Thus, classification rules generated on the basis of APU subjects can successfully classify APL subjects with high accuracy and the discriminant function variable was very significant (*P* ≤ 0.0001) on the excluded APL population (Table 3.2).

#### Classification of AEX subjects on the basis of non-AEX subjects

The discriminant was formed on CON (n = 619) versus non-AEX (n = 238) subjects. The CON subjects were 89.5% and the non-AEX subjects were 92.0% correctly classified on the basis of 24 factors; stepping was once more permitted. The excluded AEX subjects (n = 109) were 89.9% correctly classified. Thus, classification rules generated on the basis of non-AEX subjects can successfully classify AEX subjects with high accuracy and the discriminant function variable was very significant on the excluded AEX population (Table 3.3).

#### CON versus entire ADD population

The discriminant was formed on CON (n = 619) versus all ADD (n = 347) subjects. The CON subjects were 88.7% and the ADD were 94.5% correctly classified on the basis of 27 factors where stepping was permitted. By the ‘prospective’ jackknifing process, 87.9% of the CON and 93.7% were correctly classified, for an overall average success of 90.0%. This indicates that discriminant analysis based upon CON and the entire ADD population has potential for prospective classification utilization.

#### Ten split-half replications of CON versus ADD

To directly address the above classification robustness as suggested above, the entire CON plus ADD population was randomly split into two halves, a total of ten times. Each one of the ten training-sets of randomly selected CON and ADD subjects was used to create a diagnostic discriminant rule to be tested upon a separate test-set, exempted from classification rule formation. The training-set derived classification rules were then tested on each corresponding test-set. For each of the ten spit-half trials, subject membership in either the training or test sets was blindly determined by a random number generator. Results are shown in Table 4. The test-set CON subjects were correctly identified 86.75% on average (range 83.65–90.07%). The test-set ADD subjects were correctly identified 88.49% on average (range 83.15–91.77%). Moreover, when the discriminant score variables generated by the training-set were passively created and evaluated for each corresponding test-set subject, the test-set CON versus ADD t-test results for the significance of the discriminant function variable as applied to the test-set were highly significant for all 10 replications at *P* ≤ 0.0001.

**Table 4:** Ten split-half replications of full population

Part 1: Number of subjects in training and test sets and top five factors chosen per trial							
Trial	Number of training set subjects	Number of test set subjects	Number of factors used	Top five factors chosen			
1	462	504	20	13, 4, 1, 12, 40			
2	494	472	20	13, 4, 12, 1, 3			
3	512	454	23	13, 12, 4, 1, 2			
4	475	491	18	13, 3, 12, 1, 2			
5	480	486	20	13, 4, 2, 12, 7			
6	487	479	20	13, 12, 4, 9, 3			
7	479	487	23	13, 1, 4, 12, 2			
8	492	474	25	13, 4, 1, 12, 3			
9	497	469	21	13, 4, 12, 2, 7			
10	488	478	17	13, 4, 12, 3, 7			
Part 2: Ten test set classification accuracies and t-test results							
Trial	Num CON	% CON	Num ADD	% ADD			
	Correct	Correct	Correct	Correct	t	df	*P*
1	285/336	84.82	150/168	89.29	19.76	502	0.0001
2	273/311	87.78	195/218	85.71	18.55	470	0.0001
3	252/296	85.14	145/158	91.77	20.34	452	0.0001
4	275/316	87.03	158/175	90.29	21.96	489	0.0001
5	277/318	87.11	151/168	89.88	20.68	484	0.0001
6	264/307	85.99	155/172	90.12	19.49	477	0.0001
7	274/314	87.26	155/173	89.60	22.13	485	0.0001
8	272/302	90.07	155/172	90.17	20.90	472	0.0001
9	258/291	88.66	148/178	83.15	19.18	467	0.0001
10	261/312	83.65	151/166	90.96	20.32	476	0.0001
Mean		86.75		88.49			

*Num* number of, *CON* normal control, *ADD* attention deficit disorder, *t* t-test, *df* degrees of freedom, *P* probability value. Results are the number and percent of correctly classified test set subjects; t values are determined for each test set using the corresponding training-set-developed discriminant function scores

The first factor chosen in each case was Fac13. The ranked usage of the nine factors utilized across the 10 replications was: Fac13 (10x), Fac12 (10x), Fac9 (9x), Fac1 (6x), Fac3 (5x), Fac2 (5x), Fac7 (3x), Fac9 (1x), and Fac40 (1x).

Thus, there is high and consistent success in subject classification and in creation of a significant discriminant function variable across 10 split-half replications where subject grouping as within the training or test set was not determined by elements of subject’s respective clinical histories but was chosen on a truly random basis.

#### CON versus ADD across age subgroups

Discriminant analysis was performed in age limiting analysis of subpopulations 2–8 years (221 CON v. 106 ADD subjects), 8–14 years (348 CON vs. 169 ADD subjects), and 14–20 years of age (49 CON vs. 70 ADD). Analysis of the 2- to 8-year-old subjects demonstrated a significant Wilk’s lambda of 0.366 (F = 25.1; df 21,305; *P* ≤ 0.0001). A high jackknifed classification was also achieved (CON 199/221, 90.0% correct and ADD 96/106, 90.6% correct). Analysis of the 8- to 14-year-old subject groups demonstrated a significant Wilk’s lambda of 0.216 (F = 74.7; df 24,492; *P* ≤ 0.0001). A high jackknifed classification was once more achieved (CON 337/348, 96.8% correct and ADD 162/169, 95.9% correct). Analysis of the 14- to 20-year-old subject group demonstrated a significant Wilks lambda of 0.090 (F = 59.9; df 17,101; *P* ≤ 0.0001). A high jackknifed classification was once more achieved (CON 49/49, 100% correct and ADD 68/70, 97.1% correct).

Thus, classification based on age range limited subgroups manifest better success than for analyses encompassing the entire age range.

#### Classification of autism spectrum disorder (ASD) subjects on the basis of CON versus ADD rules

To explore the possibility that the apparent ability of CON versus ADD discriminants described above might simply detect any non-control subject, we made use of 430 previously studied [62] children with ASD, ages 2–12 years, whose data had been similarly de-artifacted and formed into 4416 coherence variables. The current 40 ADD factors were passively, canonically created on all ASD subjects, using currently created ADD factor loading rules, to permit comparison of ASD subjects with the current CON and ADD samples. Discriminant analysis (limited to ages 2–12) was performed on 569 CON and 237 ADD subjects. CON subjects were 91.2% (519/569) and ADD 93.7% (222/237) correctly classified. Discriminant rules created by the above 2- to 12-year-old CON plus ADD samples as a training-set was, however, only 30.7% (133/430) successful on the separately collected ASD population (treated as a passively classified test-set). These data illustrate that the current study’s ADD derived rules characterize only a fraction of ASD subjects as having ADD, which shows that CON-ADD defined classification rules do not necessarily assign all non-control subjects to the ADD group. It is notable, however, that, in general, some 30–50% of ASD patients may also manifest attentional disorder [96]. However, we did not have adequate historical information on the ASD population regarding concurrent attentional disorder to determine whether current CON-ADD-based classifiers might actually have correctly detected ADD characteristics within 30% of the ASD population.

#### Coherence loadings upon ADD factors

**Fig. 3:**
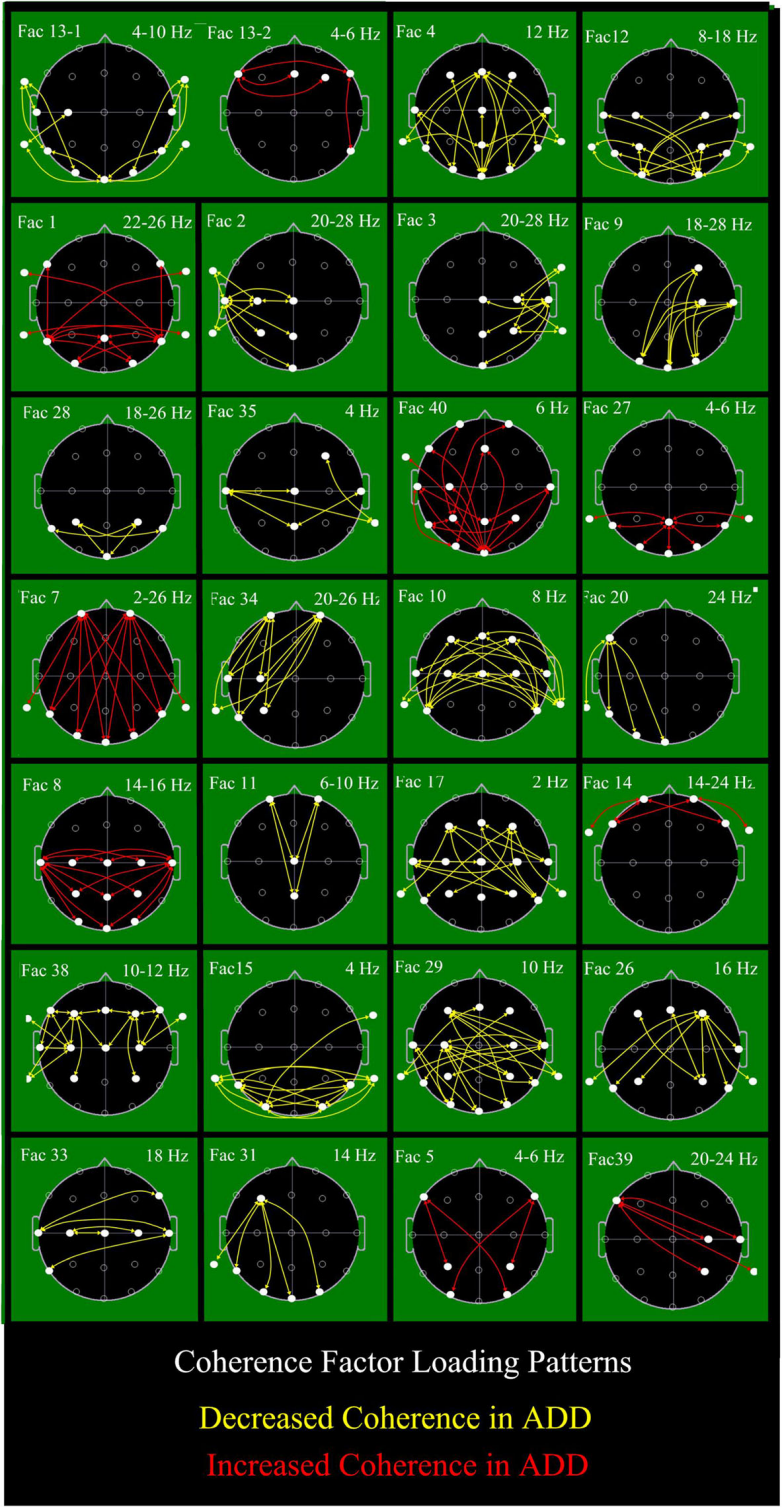
EEG coherence connectome. Twenty-seven factor loading patterns are illustrated, each within a rectangular black outlined box. The schematic black background heads are shown in vertex view, nose above, left ear to the left, and occiput below. White dots signify electrode positions (see Fig. 1). Each line represents the coherence between the electrodes at beginning and end of the line. Colored straight and curved lines signify factor loadings that either were reduced (yellow) or increased (red) for the attention deficit dirorder (ADD) group. Lines represent the top 15% loadings for the illustrated factor. Factor designation is shown to the top left and involved frequency(ies) is (are) shown to the top right of each box. These 27 factors were those utilized for the discrimination between the CON and entire ADD population (see text, Results)

Figure 1 shows electrode locations utilized in this study and their traditional names. Figure 3 shows the coherence loadings on each of the 27 factors chosen by stepwise discriminant analysis for the successful CON versus ADD analysis described above. In Fig. 3, colored lines indicate electrode coherence pairs and their color signifies coherence change relative to the ADD-group; red indicates increased and yellow decreased coherence for the ADD group as compared to the CON group. Similar factor loading displays have been used in other studies [62, 86–90] to graphically illustrate the most important coherence loadings upon a given factor by identification of the coherence loadings with the highest values per factor and additional display of all other coherence loadings that achieve within 85% or more of the highest loading value on the factor. Note that factors are displayed in Fig. 3 by the order of selection by the discriminant analysis. All schematic heads are in ‘neurological view’ , i.e., nose above and left ear to image left. The first chosen Factor 13 requires two head images as it manifests both decreased (Fac13-1) and increased (Fac13-2) coherence. All remaining 26 factors manifest only a single direction of coherence difference for the ADD population. Within the 28 head images of Fig. 3, 19 loading patterns indicate lower coherence and nine indicate higher coherence for the ADD population. Thus, reduced connectivity predominates in ADD although there are discrete factors also manifesting increased coherence in ADD.

It is important to emphasize that functional connectivity need not precisely follow anatomical connectivity. Functional connection between two spatially disparate cortical regions may be modified by many factors including those that influence synaptic coupling along the axonal pathway(s) that connect regions. There is also the possibility that two regions may be ‘connected’ by more than one neural pathway and that a shift of efficiency among the connecting pathways may alter net degree of connectivity [97].

As regards spectral bands, delta spectral activity is associated with 2, theta with 9, alpha with 8, and beta with 15 factors. There is a trend for reduced coherence in factors covering faster EEG spectral bands and increased coherence in factors covering slower spectral bands but proportional differences do not reach statistical significance by Fisher’s exact test.

By visual estimate (Fig. 3), the temporal regions are involved in 25, parietal in 17, central in 16, occipital in 15, and frontal in 16 factor loading patterns. Overall, 20 loading patterns are mostly symmetrical and bilateral whereas 8 are mostly asymmetrical or regional. Absent from these loadings are the typically chosen, linear left-right and front-back patterns selected for analysis in other coherence studies. Also note that the single most significant and utilized factor, Fac13, involved subtemporal electrodes (e.g., FT9, TP9, FT10, TP10) that have been excluded from most published analyses and yet 20 of the 28 head images in Fig. 3 involve coherence loadings involving at least one subtemporal electrode.

## Discussion

Following an extensive process to minimize/eliminate eye blink and muscle artifact, 4416 coherence variables were created per subject. The large 4416 variable by 966 subject input data matrix was successfully reduced by PCA, yielding 40 factors accounting for slightly more than 50% of the total variance (or information). This 110:1 data reduction to 40 factors avoided the need for up-front ‘educated variable selection’. Data reduction was solely guided by the underlying, intrinsic data structure of the numerous coherence variables. As can be seen in the factor coherence loading map (Fig. 3), the typically chosen pattern of left-to-right and front-to-back coherence electrode pairs is essentially absent.

When the CON and ADD subjects are compared by two group F test (Table 2), seven of the 40 factors show a significant difference at the *P* ≤ 0.0001 level. Despite this large number of highly significant factors, the observation of Sellke at al. [98] that “…*P values are often incorrectly viewed as an error probability for rejection of the hypothesis or, even worse, as the posterior probability that the hypothesis is true*” was heeded. Findings of this study are based not only upon significance (*P*) levels but also require subject classification success resulting from a multivariate discriminant processes and, where possible, the strong reliance upon estimates of prospective classification success by means of ‘left-out groups’ as in jackknifing and/or split-half replication.

At the time of initial study design, it was assumed that major findings would ultimately be based primarily upon a ‘pure’ population of subjects, namely ADD subjects off medications and without concurrent associative and/or causative symptomatology and/or neurologic diseases. Subjects taking medications (MED) and/or with mild (APL) or more definite (AEX) associative illness were, however, also identified, so that the presence of these presumed modifying influences of medications and/or associated symptoms might be explored. That these factors might have a minimal impact upon the physiology of identified clinical subgroups became evident early in the analytic process.

At the start of the discriminant analyses it was first verified that, if all 40 factors were forced into the CON (n = 619) versus APUU (n = 90) discriminant analysis, these two groups could be significantly separated (*P* ≤ 0.0001); indeed, the groups were strongly separated (91.1% correct for each). However, the remainder of the ADD population of 257 subjects in the MED, APL, and AEX subgroupings (acting as a left-out test-set) were correctly classified as ADD by the CON versus APUU discriminant process at the 87.55% level. Thus, classifiers based upon the purest ADD subpopulation nevertheless correctly classified the remaining ‘less-pure’ subjects with corresponding high accuracy. This suggests that medications and associated diseases might have little effect upon the coherence pattern associated with attentional disorders. To explore this, discriminant analysis was utilized to study each of these potentially confounding factors. The training sets were CON versus the target group (‘no MED’ or APU or ‘not AEX’) and the test set the opposing ADD group (MED, APL, or AEX). As shown in Table 3, in every case, the test set was well classified on the basis of the corresponding training set classification rules; and the discriminant function variable was also highly significant on each test set.

Thus, it would appear that the CON versus ADD group difference is driven by a connectivity difference primarily attributable to the attentional disorder itself and is only minimally influenced by medications and/or associated clinical diagnoses/symptoms. Consequently, split-half replication was undertaken on the entire CON versus ADD population for two reasons. First, to search for the possibility that there might be random groupings that might fail test-set replication thereby potentially identifying unsuspected, unique population subgroups, and secondly to determine prospective CON versus ADD classification robustness as a necessary prelude to the possibility of an EEG coherence-based ‘diagnostic’ test for ADD. As shown in Table 4, all 10 replications manifested high test-set classification success and highly significant test-set discriminant function difference. No evidence was found to suggest hidden, aberrant subpopulations within the current data set. It is also apparent that the 10 successful split-half replications might be adequate to justify consideration of EEG-based coherence data as having strong potential for a diagnostic test of ADD. However, as has been previously discussed in our study of ASD [62], clinical patients are seldom referred just to confirm that they are either neurotypical or have ADD.

As a partial test of our current ADD classifiers on an alternative clinical group, we formed ADD coherence factors on a previously studied ASD population and determined that 30% were classified as ADD. This might be a classification error but is more likely consistent with the published observation that some 30–50% of patients within general ASD population have ADD symptoms [96] despite the fact that DSM-5 indicates that once the ASD diagnosis has been made one should not also diagnose ADD. However, this ADD-ASD interaction – accurate or in error – constitutes but a partial example of the complexity of establishing a clinical diagnosis by a procedure such as EEG spectral coherence. For example, the current EEG coherence-based discriminant must be extended beyond the CON versus ADD dichotomies. Patients are seldom referred to establish single diagnostic possibilities, so there must be an exploration of EEG coherence findings within numerous other relevant clinical entities such as bipolar disorder, psychosis, global developmental delay, developmental dysphasia, dyslexia, epilepsy, closed head injury, and many more. Therefore, a hierarchical classification strategy must be developed in order to form a truly useful diagnostic tool. Finally, however, it is to be noted that, despite statements by others that ADD diagnosis is now possible by EEG [69], there are no well-known academic centers currently employing EEG-based clinical studies for this purpose. Further, one might rightly question whether clinicians really need a neurophysiological laboratory test to establish ADD.

It is notable that coherence factors evidence greater age subgroup classification success than observed within the whole group analysis despite statistical removal of age effect from the 40 factors prior to analyses. The finding of better age subgroup analysis indicates that the effect of age upon the 40 factors is non-linear; the previous ‘removal’ of age effects by regression being a linear process having no impact upon non-linear effects, which appear to persist. Implications of this observation are that multiple age subgroup classification functions should be considered when forming classifiers for clinical application; Helgadottir et al. [69] make the same observation.

At the moment, the most significant findings of the current study relate to the strong EEG coherence factors and the complex factor loading patterns, or ADD ‘connectome’ , they illuminate (Fig. 3). To start, the complex ‘connectome’ pattern made manifest by EEG coherence factors loading patterns appear able to facilitate discrimination between CON and ADD group subjects’ independent of the presence and/or absence of potentially complicating clinical factors (medications and common coexisting syndromes) that were initially felt within our group to constitute confounding variables. Or stated another way, attentional problems seen in association with other discrete syndromes, are associated with the same patterns of altered connectivity observed in ‘pure’ ADD.

As speculated by Pascual-Leone [99], the ability to sustain focused attention constitutes, along with spoken language, a crucial human strength and forms an important part of the skill-set responsible for the dominance of our species. Therefore, the discovery of a complex and extensive EEG coherence ‘connectome’ uncovered by study comparing subjects with and without disorders of attention is not surprising.

As to the specific detail, reduced EEG connectivity prevails in ADD but increased coherence is also prominent within some loading patterns. As noted in the Introduction, MRI connectivity studies demonstrate both decreased and increased connectivity as are manifested by EEG measures.

Factor 13, by far the most statistically significant factor (Fig. 3), was also the first chosen by all discriminant analyses. The Factor 13–1 loading pattern indicated a strong and mostly symmetrical disconnection between and within temporal and occipital regions bilaterally. In contrast, the Factor 13–2 image showed augmented connectivity between the left lateral frontal and the central, right, and right lateral frontal regions. The right lateral frontal region also showed increased connectivity with the right posterior temporal region. Attempts to explain the clinical/neurocognitive meaning of the patterns delineated are largely speculative. For example, the reduced occipital-temporal coherence made manifest by Factor 13–1 might signal a mild visual agnosia akin to the ‘psychic blindness’ first reported by Kluver and Bucy [100]. Alternatively, it might indicate mild visual disconnection between the dorsal lateral frontal region and temporal memory regions with visual cortex possibly signaling mild associated visual working memory dysfunction as first explained by Smith et al. [101]. Additionally, it remains possible that neither speculation may prove to be correct when and if actually evaluated.

It may be that all patterns of disconnection constitute defects of communication between and among brain regions, resulting in altered/diminished cerebral processing as shown in many factor loading images. However, regions of increased connectivity could represent either the brain’s attempts at ‘compensation’ or could represent additional interference within and among normal centers of neuronal processing. Some regions of increased connectivity could also reflect inability to suppress interfering and distracting inputs. Since factors are represented by single variables it should be possible to explain the meaning of coherence factors by correlations with cognitive and performance variables obtained from CON and ADD populations. However, ‘attention’ has very many attributes, including, as examples, adequate ‘attention duration’ , ability to ‘localize’ relevance within multiple inputs, ‘resistance to interference’ , ability to ‘shift and return’ , and overall ‘cognitive skill and/or processing speed’. Our population does not contain standardized neuropsychological testing for all subjects and therefore a further quest for factor meaning will await results of future studies where neuropsychological tests are extended to assess various facets of attention.

Without detailed behavioral and psychometric study of ADD subjects under neurophysiological investigation, inferring cognitive dysfunction solely based upon locations of the two regions manifesting altered connectivity is likely prone to error. It would be best that direct neurocognitive evaluation be undertaken to explore ‘meaning’ of factor patterns. Such assessment should be undertaken other than during neurophysiological study given the possibility for testing induced EEG contamination (e.g., by movement, muscle and/or ocular artifact); such studies are in the planning phase in this laboratory.

Although the need for an EEG-based diagnostic test for ADD can rightly be questioned, the quantitative nature of the 10 strongest factors and/or the highly significant canonical discriminant function scores might serve another potentially useful role. Changes in a single subject’s position along the factor score and/or discriminant function axis before and after a clinical intervention could provide a quantitative neurophysiological index of functional brain change related to such intervention, with the discriminant function variable constituting a possible ‘biomarker’. It is not impossible that change in neurophysiological function might precede detection of positive behavioral change and thereby avoid premature abandonment of potentially positive pharmacologic or neuro-behavioral interventions.

## Conclusion

EEG spectral coherence data gathered on a population of neurotypical controls and attentionally disordered subjects demonstrates an unanticipated, complex pattern of altered cortical connectivity. Discriminant function analysis, contrasting controls and subjects with attention disorder, demonstrates a very significant group difference that survives10 randomly generated split-half replications. Moreover, the coherence-based classification success is little altered by the presence or absence of medications and common coexisting conditions within the attention-disordered group. This, in turn, suggests that the spatial pattern of altered coherence remains dominant and unchanged in the face of variables that may alter clinical presentation.

The large subject volume and consistency of results suggest that such data could become the basis of diagnostic testing. However, it is seems more likely, at this time, that the discriminant function variables as well as the original coherence-based factors as variables might best serve as objective means for confirmation of attentional disorder and/or quantitative indices of change when derived from EEG data obtained over time, perhaps before and after therapeutic interventions of any sort.

A potential clinical application could arise from the ability to quantitatively impose previously derived ADD coherence ‘connectomes’ on patients with other primary diagnoses. As described in the Results, some 30% of an ASD population were also classified as ADD. It is well known that ASD subjects, as a group, do poorly with stimulant medications [102, 103]. It is speculated that limitation of stimulant medications to those ASD subjects with positive identification by the physiologically based ADD coherence connectome might enhance the probability for a positive clinical response.

As coherence can be taken as a measure of functional brain connectivity, the connectome pattern shown by the coherence factor loadings (Fig. 3) demonstrates, surprisingly, that there are widespread and complex regions of altered cortical connectivity with many more regions implicated than typically assumed, with no single region predominant. Although reduced connectivity prevails, regions of increased connectivity are also clearly evident. Specific functional ‘meanings’ of the coherence patterns elucidated by the factor loading images must await studies involving direct correlations between psychological variables and factor scores.

Overall, attentional disorder is a widespread syndrome with potentially devastating impact upon daily function and is often difficult to successfully treat without secondary complication(s). The complex, spatially dispersed, and across-subject consistency demonstrated in the current study is in accord with attentional disorder constituting a serious, complex, and primary disorder of brain function.

As regards ‘diagnostic use’ of the attentional disorder EEG coherence connectome, the data herein show that the current ADD connectome very accurately and reliably identifies patients with clinically manifest disorders of attention. However, as is also made manifest by the ASD results obtained, 30% of subjects securely identified as having ASD by a previously created ASD connectome were also identified as having attentional issues by the newly derived ADD connectome. Application of both the ASD and ADD connectomes were needed to clarify the assumed coexistence of attentional disorders and ASD in the same subject(s). By this example, it is clear that, whereas the current connectome accurately identifies attentional disorders, it cannot exclude co-existence of other psychiatric disorders that concurrently manifest attentional problems. Additional connectomes, specific to a number of common psychiatric disorders, must also be developed, and their interactions evaluated, prior to EEG assuming a ‘diagnostic’ role in psychiatry; such works are in progress.

## Abbreviations

ADD: Attention deficit disorder group
ADHD: Attention deficit hyperactivity disorder
AEX: Extreme subgroup
ApEn: Approximate entropy (complexity)
APL: ADD-plus subgroup
APU: ADD-pure subgroup
ASD: Autism Spectrum Disorder
BCH: Boston Children’s Hospital
BMDP: Bio-Medical Data Package, a statistical software package
CON: Neurotypical control group
df: Degrees of freedom
dACC: Dorsal anterior cingulate cortex
DNL: Developmental Neurophysiology Laboratory
DSM-IV: DSM-5, Diagnostic and Statistical Manual, Editions IV and 5
EEG: Electroencephalogram, electroencephalography, electroencephalographic
MED: Medicated sub-group
MRI: Magnetic resonance imaging
PCA: Principal component analysis
